# Iranian Nurses' Experiences of Moral Distress in Paediatric Wards: A Qualitative Study

**DOI:** 10.1002/nop2.70716

**Published:** 2026-07-30

**Authors:** Narges Rahmani, Majedeh Nabavian, FatemehSadat Seyed Nematollah Roshan, Hossein Alipour

**Affiliations:** ^1^ Comprehensive Health Research Center, Bab.C. Islamic Azad University Babol Iran; ^2^ Department of Nursing, TeMS.C. Islamic Azad University Tehran Iran; ^3^ Mazandaran University of Medical Sciences Sari Iran

**Keywords:** moral distress, nursing ethics, paediatric nursing, qualitative research

## Abstract

**Background:**

As direct and continuous providers of patient care, nurses are more susceptible to various forms of psychological stress within the healthcare environment compared to other healthcare professionals. Nurses in paediatric wards, who maintain constant contact with children and their families, are particularly vulnerable to moral distress. This study aimed to explore the perceptions and experiences of Iranian nurses regarding moral distress in paediatric settings.

**Methods:**

This qualitative study employed inductive content analysis, conducted between 2023 and 2024. Data were collected through semi‐structured individual interviews and analysed following Graneheim and Lundman's approach. Twelve participants experiencing moral distress were selected via purposive sampling from the Babol and Tehran Universities of Medical Sciences.

**Results:**

The findings identified four primary categories and eight subcategories: moral distress related to colleagues (doctors and nursing colleagues); moral distress related to parents (conflict with children's rights and distrust of nurses); moral distress related to organizational factors (understaffing and workload undermining holistic care and professional ethics; and inadequate equipment and resources compromising care and fostering dishonesty); and psychological tensions following moral distress (mental conflict involving the cognitive and emotional burden of moral compromise; and helplessness and despair in the face of systemic failure). Rather than representing isolated individual experiences, these findings illustrate that moral distress in paediatric nursing is deeply rooted in the structural and relational conditions of healthcare settings. The results highlight that repeated exposure to ethical conflicts can erode professional values, impede the delivery of holistic care, and intensify emotional suffering. Consequently, this study contributes to a deeper understanding of moral distress as a systemic phenomenon requiring organizational intervention rather than merely an individual coping response.

**Conclusion:**

The findings underscore the necessity for healthcare managers and policymakers to implement systemic mechanisms, such as specialized educational programmes and workshops, to mitigate moral distress among nurses. Furthermore, identifying the inherent stressors in paediatric nursing and reducing nurses' exposure to moral dilemmas are essential steps toward safeguarding ethical practice and enhancing care quality. This study contributes by shifting the focus from merely describing moral distress as a set of themes toward understanding it as a critical indicator of organizational strain and ethical vulnerability in paediatric nursing.

## 1 Introduction

Moral distress is a profound subjective experience that occurs when an individual's moral integrity is compromised, or when they feel unable to act in alignment with their core values and professional commitments despite earnest efforts to achieve an ethical outcome (Bagheri et al. 2023). According to the seminal framework by Jameton and Jackson (1984), nurses in clinical settings encounter three distinct ethical challenges: moral uncertainty, moral dilemmas, and moral distress. The latter is specifically characterized by situations where ‘someone knows the right thing to do, but organizational constraints make it almost impossible to follow through’ (Giannetta et al. 2022). Although initially conceptualized within the nursing profession, contemporary research indicates that moral distress serves as a broader response to diverse moral conflicts experienced across various healthcare disciplines (Fourie 2015).

Fundamentally, moral distress manifests as an emotional and psychological tension arising from the conflict between external constraints—such as institutional limitations—and professional responsibilities, which ultimately prevents nurses from exercising their clinical judgement and upholding ethical values (Tajalli et al. 2021). This distress may develop progressively as the care delivered increasingly diverges from the nurse's personal and professional ethical standards (Banazadeh and Rafii 2021). Previous studies suggest that these ethical challenges are driven by a complex interplay of personal factors (including values, competence, and experience), organizational barriers (such as resource scarcity and workplace culture), professional subordination, cultural nuances, and educational gaps (Blackwood and Chiarella 2020). Consequently, even when possessing advanced critical thinking skills, nurses may encounter restrictions imposed by hierarchical structures, organizational policies, or interprofessional conflicts, all of which culminate in moral distress (Chiappinotto et al. 2024).

## 2 Background

The literature regarding moral distress within healthcare systems and its ramifications for professionals has expanded considerably. Research typically categorizes moral distress into three distinct levels: patient‐related, unit‐related, and system‐related phenomena (Giannetta et al. 2022; Prompahakul and Epstein 2020; Villa et al. 2021), suggesting that such disturbances are prevalent across diverse clinical environments (Delfrate et al. 2018; Lamiani et al. 2020; Lazzari et al. 2020). Nurses are particularly susceptible to these ethical dilemmas due to the intense physical and psychological pressures inherent in their roles, including heavy workloads, staffing shortages, fatigue, and frequent exposure to end‐of‐life decision‐making. Such conditions can impede a nurse's ability to act in accordance with their moral judgement, thereby exacerbating distress (Dodek et al. 2016).

Paediatric nursing, in particular, demands a unique synthesis of scientific expertise and ethical acumen, necessitated by continuous interaction with vulnerable children and their families (Russell 2012). Providing effective ethical care in this field requires a commitment to holistic development—addressing the child's physical, psychological, and social needs—while maintaining communicative empathy and adhering to international standards, such as the UN Convention on the Rights of the Child (UNCRC) (Khalili et al. 2023). Despite these rigorous standards, a Swedish study revealed that fewer than one‐third of paediatric nurses perceived the ethical quality of their practice environment positively (Ventovaara et al. 2021). Furthermore, research consistently links ethical decision‐making capacity to both job satisfaction and the prevalence of moral distress (Koskenvuori et al. 2019).

While the American Nurses Association emphasizes the implementation of advanced systematic processes for paediatric care (Behbodi et al. 2018), nurses often struggle to execute ideal care plans due to resource limitations, complex family dynamics, or the severity of the child's clinical condition (Hendrick 2011; Rabiee et al. 2012). Paediatric care presents distinct challenges; for instance, distress is intensified when nurses must administer painful treatments or navigate situations where parents withhold medical truths from their children (Bartholdson et al. 2015).

The consequences of moral distress are multifaceted and detrimental to nurses, patients, and healthcare systems alike. It can compromise patient care, potentially hindering the healing process and prolonging hospitalizations (Molazem et al. 2013; Tahmasebi et al. 2022). For nurses, sustained distress elicits severe emotional responses—such as anger, despair, and guilt—alongside somatic symptoms like headaches and sleep disturbances (Trotochaud et al. 2015). Moreover, it can erode professional confidence and agency; notably, 81% of nurses in intensive care units reported feeling powerless due to moral tension (Naboureh et al. 2021). This sense of discomfort and anxiety arises when nurses confront situations beyond their control, preventing them from acting in the best interest of their patients (Ventovaara et al. 2021).

In the Iranian context, studies addressing decision‐making and moral distress within paediatric hospitals remain scarce. Much of the existing local research has focused narrowly on paediatric intensive care units (Behbodi et al. 2018; Tahmasebi et al. 2022; Ventovaara et al. 2021), which may operate under distinct clinical paradigms. Furthermore, most prior investigations have employed quantitative methodologies; consequently, there is a lack of qualitative exploration regarding the lived experiences of nurses facing moral distress in Iran. Therefore, the present study aims to comprehensively explore the experiences of paediatric nurses regarding moral distress using an inductive content analysis approach.

## 3 Methods

### 3.1 Study Design and Approach

This study employed a qualitative descriptive design, utilizing conventional content analysis to explore the lived experiences of moral distress among nurses in paediatric hospital settings. Qualitative research is uniquely suited for the in‐depth exploration of individual experiences within their natural contexts—an approach driven by the study's specific aims and the phenomenon under investigation (Rahmani et al. 2021). Given the nascent and fragmented nature of existing knowledge regarding moral distress, particularly within the Iranian paediatric nursing context, an inductive conventional content analysis approach was selected as the most appropriate methodology. As conceptualized by Wildemuth (2016), content analysis serves as a systematic method for deriving replicable and valid inferences from data, thereby facilitating knowledge generation, the development of new insights, factual representation, and practical guidance. Recognizing moral distress as a complex, multifaceted phenomenon characterized by intricate thoughts, emotions, and internal conflicts, qualitative content analysis provided a robust and flexible framework. This methodology facilitates a nuanced interpretation of lived experiences and the systematic identification and categorization of emergent patterns within the dataset (Graneheim and Lundman 2004). The overarching objective of this study was to achieve a profound understanding of the ethically burdensome situations encountered by paediatric nurses, including navigating complex treatment decisions, managing interactions with distressed families, and addressing constraints imposed by resource limitations.

### 3.2 Epistemological Stance and Researcher Reflexivity

This research is anchored in an interpretivist epistemology, which posits that reality is socially constructed and that understanding is achieved through an exploration of the meanings participants ascribe to their experiences (Lincoln and Guba 1985). This philosophical underpinning informed our approach to uncovering the subjective and context‐dependent nature of moral distress. Throughout the research process, the team rigorously engaged in reflexivity. Comprising individuals with prior clinical experience in paediatric wards, the research team actively acknowledged and critically examined their pre‐existing assumptions and potential biases. To systematically mitigate the influence of personal perspectives on data collection and analysis, reflexive memos were meticulously prepared prior to interviews and maintained throughout the iterative process of coding. These reflections were regularly discussed among all team members, ensuring a clear and conscious demarcation between the researchers' own experiences and the participants' narratives. This collaborative and sustained engagement with reflexivity was critical in maintaining analytic openness, minimizing undue influence on data interpretation, and ultimately enhancing the credibility and trustworthiness of the study's findings.

### 3.3 Participants and Research Context

Participants were recruited through a purposive sampling strategy, selecting nurses working in paediatric wards who had self‐identified as experiencing moral distress. Those who were willing to participate were selected, while those who were unwilling or unable to complete the interview were excluded. Regarding patient and public involvement, patients or members of the public were not involved in the design, conduct, interpretation, or dissemination of this study; however, professional nurses participated as key informants, contributing their lived experiences to the qualitative inquiry. A maximum diversity sampling strategy was subsequently employed to include nurses representing a wide range of genders, clinical wards, and hospital types (as detailed in Table 1), thereby ensuring a rich and multifaceted dataset that captured diverse perspectives.

**TABLE 1:** Demographic and clinical characteristics of participants (*N* = 12).

Characteristic	Category	*n* (%)
Gender	Male	4 (33.3)
	Female	8 (66.7)
Marital status	Married	10 (83.3)
	Single	2 (16.7)
Education	Bachelor's	10 (83.3)
	Master's	2 (16.7)
Hospital ward	Emergency	5 (41.7)
	Surgery	3 (25.0)
	Infectious	2 (16.7)
	Oncology	2 (16.7)
City	Babol	4 (33.3)
	Tehran	8 (66.7)
Hospital type	Government	10 (83.3)
	Private	2 (16.7)

Data were gathered through in‐depth, semi‐structured, individual interviews conducted in a quiet environment at the participants' workplaces in Tehran and Babol. Following the procurement of comprehensive informed consent, interviews commenced with broad, open‐ended questions designed to encourage participant elaboration and rich description. Examples include: ‘Please describe your experiences during a typical shift in the paediatric ward,’ ‘What ethical issues have you encountered while caring for a child?’, and ‘What factors contributed to your moral distress while caring for a child?’ This open‐ended approach allowed participants the freedom to elaborate, providing significant depth to their accounts. The interview process was subsequently guided by the research aims, incorporating clarifying and probing questions (e.g., ‘Please explain further,’ ‘What do you mean by that?’) to ensure a comprehensive exploration relevant to the study's objectives.

### 3.4 Data Saturation and Analysis Process

The final sample size was determined by the principle of data saturation, defined as the point at which additional data collection yields no new relevant concepts, categories, or insights regarding the research questions (Davies and Logan 2021). Data collection was conducted iteratively, occurring concurrently with ongoing analysis and coding. A total of 12 nurses were interviewed; saturation was empirically achieved by the tenth interview, as no new significant categories or codes emerged from subsequent discussions. To rigorously confirm saturation, the coding and categorization process was meticulously reviewed and verified by the entire research team, including the principal investigator and the research assistants who performed the initial coding. The final two interviews served to solidify this observation, yielding no novel insights and reinforcing the established categories. Based on the research team's collective consensus regarding data redundancy and thematic saturation, data collection was concluded. This systematic, team‐based approach to achieving and confirming data saturation, coupled with the iterative nature of the analysis, significantly contributes to the robustness and trustworthiness of the findings. Interviews, each lasting between 40 and 60 min, were conducted individually in a quiet, private environment at the participants' workplaces (Flanagan and Beck 2024), audio‐recorded, and immediately transcribed verbatim.

### 3.5 Analysis Team and Roles

Data collection, initial coding, and ongoing analysis were primarily conducted by three members of the research team (Authors 1, 2, and 3). Author 1 engaged in deep immersion with the data through repeated listening to audio recordings and verbatim transcription, followed by the identification of meaning units and preliminary coding. Authors 2 and 3 collaborated closely with Author 1 during the subsequent analytical stages. Employing the constant comparative method, the team systematically compared codes across all interviews to identify recurring similarities and nuances. Codes sharing common conceptual threads were progressively grouped into subcategories. As the analysis deepened, these subcategories were further abstracted and synthesized into broader, higher‐level categories, each assigned a distinct conceptual label. For instance, codes such as ‘Patient harm due to physician's lack of technical skill’ and ‘Physician's reprimand for sharing medical information with families’ were initially grouped and abstracted into the subcategory ‘Moral distress related to physician colleagues.’ The interrelationships among these emergent categories were meticulously examined through iterative reflection and constant comparison with the raw data by Authors 1, 2, and 3. For example, subcategories such as ‘Moral distress related to physician colleagues’ and ‘Moral distress related to nursing colleagues’ were integrated to form the overarching category of ‘Moral distress related to colleagues’ (Table 2). Throughout this process, the team ensured a unified interpretation, with no significant disagreements arising regarding coding or analysis. Author 4 was responsible for the final manuscript preparation and the structured presentation of the findings. This methodological division of labor—combining rigorous, multi‐analyst analysis with focused synthesis—enhanced both the rigour and transparency of the analytical process. This study was reported in accordance with the Consolidated Criteria for Reporting Qualitative Research (COREQ) guidelines to ensure transparency and rigour in the reporting of the qualitative findings.

**TABLE 2:** Example of qualitative data analysis for moral distress related to colleagues.

Meaning units (participant quotations)	Primary codes	Sub‐category	Category
‘I was in a private hospital. I had just entered the ward. I checked the patient's chart and informed the mother that her child would only need Pedialyte. The physician pulled me aside and said, You had no right to disclose medical information to the mother. Who do you think you are?’ ‘Sometimes, when a physician lacks proficiency in a procedure like intubation but insists on performing it, I intervene to prevent potential harm. However, they often dismiss my concerns due to hierarchical differences…’	Physician's reprimand for sharing medical information with families Disregard for nurses' clinical concerns Patient harm due to physician's lack of technical skill Denial of skill deficit despite negative outcomes Psychological impact on nurse from preventable harm	Moral distress related to physician colleagues	Moral distress related to colleagues
‘A child was in the emergency room for two hours due to sexual abuse. The child's privacy was broken … but no one cared about this. I was so shocked, and I couldn't say even a word’ Some colleagues seem to have to do venipuncture by themselves… Those with a long work experience have tried more than 10 times	Disregard for patient privacy Patient and family distress due to repeated examinations Unwillingness to delegate tasks to competent colleagues Persistence in performing procedures despite fatigue Risk of physical and psychological harm to patients from repeated attempts	Moral distress related to nurse colleagues	

### 3.6 Trustworthiness and Rigour

To ensure the rigour of this qualitative study, we adhered to Guba and Lincoln's criteria for trustworthiness (Nabavian et al. 2025). Credibility was established through building strong rapport with participants, prolonged engagement during data collection and analysis, and the triangulation of data through multiple perspectives and expert reviews. Transferability was ensured through the ‘thick description’ of the research context and participant characteristics, allowing readers to assess the applicability of the findings to other settings. Dependability was facilitated by involving faculty members not directly involved in the study to review selected interviews, codes, and categories, thereby establishing a robust audit trail. Finally, confirmability was strengthened through continuous supervision and external auditing, ensuring that the findings emerged directly from the data rather than from the researchers' preconceived biases.

### 3.7 Ethical Considerations

This study was approved by the Ethics Committee of Islamic Azad University, Babol Branch (approval code: IR.IAU.BABOL.REC.1402.115). Prior to the interviews, all participants were provided with comprehensive information regarding the study's objectives, procedures, and the strictly voluntary nature of their participation. As all participants were adult registered nurses (aged 18 years or older), written informed consent was obtained from each individual before their inclusion in the study. All research procedures were conducted in strict accordance with relevant national and institutional ethical guidelines and regulations.

## 4 Results

The qualitative analysis of paediatric nurses' experiences of moral distress yielded 530 primary codes, which were systematically categorized into 4 overarching categories and 8 subcategories (Table 3).

**TABLE 3:** Categories and subcategories of data analysis for identified concepts.

Class/Category	Subclass/Specific issue
Moral distress related to colleagues	Moral distress related to doctor colleagues
	Moral distress related to nursing colleagues
Moral distress related to parents	Conflict with children's rights
	Distrust of the nurse
Moral distress related to organizational factors	Understaffing and workload: undermining holistic care and professional ethics
	Inadequate equipment and resources: compromising care and fostering dishonesty
Psychological tensions following moral distress	Mental conflict: the cognitive and emotional burden of moral compromise
	Helplessness and despair in the face of systemic failure

### 4.1 Moral Distress Related to Colleagues

Moral distress in paediatric settings emerges as a relational and systemic phenomenon, driven by the breakdown of professional collaboration and the presence of power imbalances within the multidisciplinary team. This dimension manifests through two distinct mechanisms: vertical hierarchical tension and horizontal accountability failure.

#### 4.1.1 Moral Distress Related to Physicians

Distress involving physicians reveals a profound structural imbalance in how knowledge is valued within the clinical hierarchy. Participants frequently described situations where their clinical observations were dismissed by physicians, leading to a sense of intellectual and professional silencing. For instance, when describing failed clinical interventions (e.g., M9: ‘the patient expired… I still see the child's face’), the distress was compounded by the inability to provide input. This experience illustrates a form of epistemic injustice—specifically, hermeneutical injustice—where in the nurse possesses the experiential knowledge to interpret a clinical failure but lacks the social and professional standing to have that interpretation recognized as valid. The physician's refusal to accept clinical feedback—described by participants as ‘he/she does not want to accept it because they are a doctor and we're nurses’—operationalizes this asymmetry, whereby positional power is asserted over empirical evidence.

Furthermore, this distress extends to professional disenfranchisement through disciplinary intimidation. When nurses attempted to fulfil their ethical duty of patient education, they were often met with gatekeeping mechanisms. One participant (M8) recounted being reprimanded: ‘You had no right to give medical information…’ Such experiences effectively criminalize the nurse's ethical agency, forcing a compromise between professional integrity and organizational compliance. This sense of being stripped of autonomy was reflected in the participants' self‐description as ‘robots’—a phenomenological manifestation of an environment wherein clinical judgement is rendered epistemically null.

#### 4.1.2 Moral Distress Related to Nursing Colleagues

In contrast to physician‐related distress, distress stemming from nursing colleagues arises from a breach of professional solidarity, often leading to moral isolation within the peer group. This is frequently driven by the normalization of unethical practices.

The violation of a child's privacy during sensitive procedures serves as a stark example of this collective desensitization. As one participant (M6) noted: ‘everyone was allowed to check me… I was so shocked, and I couldn't say even a word.’ The observation that ‘everyone was looking’ suggests a diffusion of responsibility, whereby the group's collective gaze de‐moralizes the act. This aligns with the concept of systemic ethical drift, wherein individual moral standards are eroded by group consensus.

Additionally, distress arises from professional hubris facilitated by fatigue, whereby colleagues may refuse assistance to maintain a defensive performance of competence. For example, M8 stated: ‘My colleague… did not let me do the child venipuncture.’ This creates a dual moral burden: the nurse witnesses preventable suffering while simultaneously feeling complicit due to the fear of breaching collegial norms. The resulting distress is uniquely relational, trapping the nurse in a moral compromise that she did not initiate but feels unable to escape without violating professional loyalty.

### 4.2 Moral Distress Related to Parents

Moral distress in paediatric nursing is uniquely complicated by the presence of parents and caregivers, creating a triadic ethical struggle between the nurse, the child, and the family. This dimension arises when the nurse's duty of child advocacy clashes with parental autonomy, often leaving the nurse navigating a precarious middle ground between clinical necessity and familial influence.

#### 4.2.1 Conflict With Children's Rights

A primary source of distress is the ethical dissonance between evidence‐based nursing practice and parental decisions shaped by cultural beliefs or varying levels of health literacy. In these instances, the nurse's fundamental obligation—to act in the ‘best interests of the child’—is frequently compromised by parental actions that pose direct risks to patient safety.

A critical example is the administration of traditional substances, such as opium, to infants to induce sedation (e.g., M6). In such cases, when nurses attempt to intervene to protect the infant, they are often met with intense familial resistance. This experience transforms the nurse from an active protector into a ‘helpless witness.’ This phenomenon exemplifies a profound ‘advocacy gap’: while the nurse possesses the clinical insight to recognize harm, they lack the systemic or legal authority to override parental autonomy in cases involving non‐life‐threatening but harmful traditional practices. The inability to bridge this gap results in significant moral injury, as the nurse is forced to witness the violation of the child's safety while being structurally precluded from intervention.

#### 4.2.2 Distrust of Nurses

Beyond direct conflicts regarding child safety, distress is generated by parental demands that undermine professional boundaries and clinical protocols. Nurses described this not merely as a struggle with ‘demanding parents’ but as an erosion of professional authority fueled by a pervasive ‘climate of fear.’

Nurses frequently experience a ‘dual‐loyalty conflict’ when parental anxiety drives requests that contravene physician orders, such as demanding unnecessary medications (e.g., M5). This situation forces the nurse to navigate the tension between clinical fidelity—adherence to medical orders and evidence‐based protocols—and the socio‐emotional pressure to satisfy family members in order to avoid interpersonal or administrative conflict.

This tension is further intensified by constant scrutiny and physical interference during clinical procedures. For instance, the fear that enforcing professional boundaries—such as asking a parent to step back during a venipuncture (e.g., M2)—will trigger formal administrative complaints creates a state of professional vulnerability. This perceived power of parents to initiate institutional repercussions leads to a state wherein the nurse's autonomy is systematically undermined. Consequently, nurses often feel compelled to prioritize ‘parental appeasement’ over clinical efficiency and patient‐centred care, resulting in a pervasive sense of compromised professional integrity and learned helplessness in the face of institutional and familial pressure.

### 4.3 Moral Distress Related to Organizational Factors

Organizational structures, policies, and resource allocation serve as macro‐level determinants of moral distress. This study reveals that for nurses, moral distress is not merely a reactive phenomenon to clinical challenges but is deeply rooted in systemic failures. These constraints foster a state of ‘moral dissonance,’ wherein professional ethical standards clash with the pragmatic, often suboptimal, realities of the healthcare environment. This category comprises two primary dimensions: (1) Understaffing and Workload, and (2) Inadequate Equipment and Resources.

#### 4.3.1 Understaffing and Workload: Undermining Holistic Care and Professional Ethics

This dimension examines how staffing shortages and excessive workloads impede the core ethical tenets of nursing, particularly the provision of holistic and compassionate care. When nurses are forced to prioritize task‐oriented efficiency over patient‐centred empathy, they experience a profound ethical compromise.

Participants indicated that heavy workloads prevent them from addressing the psychosocial needs of paediatric patients and their families, leading to significant guilt and self‐reproach. For instance, M10 expressed deep guilt over being unable to support a worried family member due to exhaustion, stating: ‘I somehow feel guilty about it… it bothers me.’ This inability to provide the level of care they deem ethically necessary illustrates the accumulation of ‘moral residue’—the enduring psychological weight that remains after a nurse has been forced to compromise their professional values due to structural constraints. This process effectively leads to an ‘erosion of compassion,’ wherein nursing is transformed from a therapeutic encounter into a mechanical, task‐driven activity.

Furthermore, the nexus between workload and patient safety is a critical driver of distress. High patient‐to‐nurse ratios were perceived to escalate the risk of medication errors, triggering an internal crisis of professional identity. One participant (M12) recounted a medication error caused by distraction, expressing profound feelings of unworthiness. Such experiences highlight how organizational failures can precipitate ‘moral injury’; here, errors are not viewed merely as transient clinical lapses but as fundamental violations of professional integrity, necessitated by an environment that renders such errors nearly inevitable. This results in deep‐seated feelings of inadequacy and shame rather than simple occupational stress.

#### 4.3.2 Inadequate Equipment and Resources: Compromising Care and Fostering Dishonesty

The second dimension involves moral distress arising from the scarcity of essential resources, such as medications and functional equipment. Such scarcity forces nurses into ‘distributive justice’ dilemmas, requiring impossible choices regarding the equitable allocation of limited resources.

Participants described how supply shortages compelled them to engage in behaviours that directly contradicted their professional commitment to honesty and transparency. For example, M11 detailed the ethical tension of being unable to provide medication to a patient from another ward due to local shortages, which led to feelings of being ‘betrayed’ by the necessity of prioritizing one patient over another. This experience reveals a profound ‘moral alienation’: when organizational deficiencies force nurses to compromise their integrity—through selective care or being forced into deceptive professional stances—they become fundamentally disconnected from the ethical foundations of their profession. Ultimately, resource scarcity does not merely impede clinical tasks; it actively undermines the nurse's moral agency.

### 4.4 Psychological Tensions Following Moral Distress

The aftermath of moral distress manifests as profound psychological tensions that permeate the nurse's cognitive and emotional existence. This category transcends mere occupational stress, representing a deeper state of psychological erosion. It is characterized by two primary dimensions: Mental Conflict (characterized by cognitive intrusion) and Exhaustion (characterized by emotional and existential depletion).

#### 4.4.1 Mental Conflict: The Cognitive and Emotional Burden of Moral Compromise

Mental conflict in this context refers to the intrusive and persistent nature of moral distress, wherein ethical dilemmas effectively ‘colonize’ the nurse's private cognitive space. This is not merely a transient state of ‘worrying’ about a patient but a profound inability to mentally detach from perceived ethical failures.

For instance, M7 described how a case of parental negligence—a child falling through a fence—led to prolonged ‘inner stress’ and an obsessive need to discuss the event. This phenomenon illustrates ‘cognitive rumination’—a hallmark of moral injury wherein the mind becomes trapped in a repetitive cycle of re‐experiencing the ethical breach. The nurse's metaphorical attempt to ‘shield the fence’ exemplifies the psychological struggle to rectify an unfixable ethical situation, creating a sustained mental burden that blurs the boundaries between professional duty and personal trauma.

Furthermore, mental conflict is intensified by interpersonal friction within clinical teams. When colleagues bypass safety protocols, nurses are forced into a state of constant hyper‐vigilance. This generates acute ‘cognitive dissonance’ between the nurse's internal professional standards and the external reality of a non‐compliant team, ultimately resulting in chronic mental fatigue and a pervasive sense of futility.

#### 4.4.2 Exhaustion: Helplessness and Despair in the Face of Systemic Failure

The second dimension, exhaustion, represents the terminal stage of moral distress, wherein nurses experience a total depletion of emotional and professional agency. This is not mere physical tiredness but a profound ‘moral disempowerment.’

This exhaustion is most evident when nurses witness repeated professional wrongdoing that remains unaddressed by the system. M11's statement—‘I read and studied all this, but it didn't help’—poignantly expresses ‘professional disillusionment.’ It signifies the realization that clinical expertise and ethical commitment are often powerless against systemic inertia and a lack of accountability. This realization precipitates a state of ‘learned helplessness,’ wherein nurses cease to believe that their advocacy can impact patient outcomes, ultimately paving the way for burnout and emotional withdrawal.

This state is further exacerbated by tragic, unpredictable adverse events, such as the sudden death of a stable neonate. The intense sense of personal culpability expressed by participants (e.g., ‘I knew myself to be the cause of the child's death’) reflects ‘existential guilt.’ Even in the absence of clinical error, nurses often internalize tragedies as personal moral failures. Such profound psychological pressure triggers a drive toward ‘professional avoidance’ (e.g., seeking leave or withdrawing from the ward), as the clinical environment transforms from a site of healing into a landscape of psychological trauma. Consequently, the psychological tension following moral distress is not merely a symptom but a transformative process that can fundamentally alter a nurse's professional identity and their capacity for compassionate care.

## 5 Discussion

While previous investigations have extensively documented the triggers of moral distress across diverse healthcare settings (Abbasi et al. 2019; Morley et al. 2021; Morley et al. 2023), the present study elucidates a complex and multifaceted phenomenon within paediatric nursing. Our findings suggest that moral distress in this specialty is not merely a reactive emotional state to clinical challenges but a structurally mediated experience emerging from the intersection of specialized clinical exigencies, rigid institutional hierarchies, and intense familial dynamics.

In the realm of professional relationships, the tension between clinical vigilance and hierarchical obedience extends prior observations in the field. While existing studies have established a robust association between reduced professional autonomy and moral distress (Bagheri et al. 2023; Giannetta et al. 2021; Papathanassoglou et al. 2012), our qualitative findings provide a more granular elucidation of the underlying mechanism: a profound structural imbalance in how clinical knowledge is valued. Participants' narratives, particularly those describing the dismissal of their clinical observations by physicians, reveal a clear manifestation of epistemic injustice (Fricker et al. 2007). Specifically, we observed elements of hermeneutical injustice, wherein nurses possess the experiential knowledge to interpret clinical shifts but lack the professional standing to have these interpretations recognized as valid within the clinical hierarchy. This ‘suffocating silence’ and the feeling of being rendered ‘epistemically null’—as reflected in participants' descriptions of being treated like ‘robots’—suggest that moral distress is compounded when professional agency is structurally undermined by positional power. Furthermore, the breach of professional solidarity among nursing colleagues, characterized by the normalization of unethical practices or a ‘diffusion of responsibility,’ highlights how systemic ethical drift can lead to moral isolation even within peer groups.

Furthermore, this study offers a nuanced refinement of the role played by family dynamics. Although prior research has identified family contact as a significant risk factor (Rabiee et al. 2012; Russell 2012), our findings challenge the reductive view that distress arises solely from parental presence. Instead, we identify a complex ‘triadic ethical struggle’ involving the nurse, the child, and the family. This struggle is driven by an intense conflict between the nurse's fundamental obligation to act in the ‘best interests of the child’ and the necessity of respecting parental autonomy, especially in cases involving culturally rooted practices or health literacy gaps. (Berdida 2023). The inability to bridge this ‘advocacy gap’ transforms the nurse from an active protector into a ‘helpless witness,’ a role that precipitates significant moral injury (Dean et al. 2019). This finding contributes to the literature by transitioning from a general understanding of ‘family‐related stress’ toward a specific framework of ethical dissonance inherent in paediatric advocacy.

Regarding organizational determinants, our research builds upon the foundational work of Challinor et al. (2020) and Hopia and Heino‐Tolonen (2019) by providing a deeper interpretative layer. While these studies established the link between staffing shortages and moral distress, our findings suggest that such shortages act as macro‐level determinants that systematically erode the possibility of compassionate, holistic care. In the specialized paediatric setting, organizational failures transcend mere task inefficiency; they induce a state of moral alienation (Sandeberg et al. 2023). As participants described the persistent inability to meet the psychosocial needs of critically ill children due to workload, they experienced a profound sense of ‘moral residue’—an enduring psychological weight resulting from the compromise of professional values. Moreover, when resource scarcity forces nurses into ‘distributive justice’ dilemmas or compels them to prioritize ‘parental appeasement’ over clinical protocols, it actively undermines their moral agency. Consequently, organizational policy must be reconceptualized not merely as a management concern but as a primary driver of professional moral injury and alienation.

Finally, our exploration of the psychological aftermath of moral distress adds a critical dimension to the existing discourse on burnout and turnover (Abbaszadeh et al. 2013; Delfrate et al. 2018; Golbach et al. 2022; Pauly et al. 2012). While previous scholarship has frequently emphasized long‐term professional attrition, this study illuminates the immediate, intrusive cognitive and emotional mechanisms that characterize the paediatric experience. The emergence of learned helplessness (Miller and Seligman 1975) was particularly evident in participants' descriptions of repeated, unsuccessful advocacy efforts, leading to a state of professional disillusionment. This is further exacerbated by the ‘mental conflict’ and ‘cognitive rumination’ described by nurses, wherein ethical dilemmas effectively ‘colonize’ their private cognitive space. The intense emotional engagement required in paediatric care creates a unique psychological vulnerability, wherein moral distress evolves from job dissatisfaction into deep‐seated existential guilt and professional avoidance (Araghian Mojarad and Shafipour 2023; Benbenishty et al. 2022; Deschenes et al. 2020; Han et al. 2023; Zalud et al. 2024).

In conclusion, these findings suggest that moral distress in paediatric nursing is a systemic and relational phenomenon rather than an isolated individual reaction. We suggest that interventions must move beyond general stress management to address the structural, hierarchical, and interpretive conditions that foster epistemic injustice, moral alienation, and learned helplessness. Addressing these systemic roots is essential to preserving the moral integrity and professional well‐being of the paediatric nursing workforce.

## 6 Strengths and Limitations

This study contributes significantly to the nursing literature by shifting the discourse from an individualistic view of moral distress toward a systemic mapping of the organizational power dynamics that shape the ethical lives of paediatric nurses. By conceptualizing how institutional failures and hierarchical pressures manifest as ‘moral injury’ and ‘professional disillusionment,’ this research refines the understanding of paediatric nursing ethics within complex, high‐stakes healthcare environments.

However, certain limitations warrant consideration. As a qualitative inquiry, the primary objective was to achieve interpretative depth rather than statistical breadth; therefore, the findings reflect the nuanced experiences of participants within the specific Iranian healthcare context. While this limits universal generalizability, it provides a high degree of contextual validity that is often missing in large‐scale, cross‐cultural studies. Furthermore, while purposive sampling was essential for capturing the intricate layers of moral distress, the focused nature of this approach may not encompass the full spectrum of experiences across all diverse paediatric nursing settings (e.g., neonatal vs. outpatient). Additionally, the current lack of culturally adapted, validated psychometric instruments specifically designed for the Iranian paediatric context precluded a quantitative triangulation of these qualitative insights—a gap that this study explicitly highlights for future scholars.

## 7 Conclusion

The findings of this study reveal that moral distress in paediatric nursing is not merely an individual emotional strain but a systemic byproduct of fragmented professional hierarchies and organizational constraints. If left unaddressed, this distress risks evolving from acute episodes into a state of chronic ‘moral alienation,’ leading to a fundamental erosion of nursing agency. This erosion does not merely affect the individual; it inevitably compromises the safety, advocacy, and quality of care provided to the most vulnerable paediatric patients.

### 7.1 Implications for Practice, Management, and Education

To move beyond reactive, individual‐focused solutions, healthcare organizations must transition toward building a robust ‘ethical infrastructure.’

For Management: There is an urgent need to shift from traditional, rigid hierarchical models toward collaborative governance frameworks that formalize and protect the nurse's role in ethical decision‐making. Managers must proactively facilitate structured channels for ‘moral dialogue,’ recognizing that safeguarding a nurse's moral integrity is fundamentally synonymous with safeguarding patient safety.

For Education: Curricular initiatives should transcend traditional clinical skill‐building to incorporate ‘moral literacy.’ Nurses must be equipped with the conceptual and communicative tools necessary to navigate the ‘triadic ethical struggles’ and the complex power dynamics inherent in paediatric care.

### 7.2 Future Research

The findings of this study invite a significant shift in the trajectory of future nursing research. First, there is a critical need for the development and validation of culturally adapted psychometric instruments in the Iranian context to capture the specific nuances of moral strain identified here. Second, the field must move beyond descriptive prevalence studies toward longitudinal and interventional research. Future studies should employ mixed‐methods approaches to evaluate the impact of systemic reforms—such as the implementation of ethics consultation services and shared‐governance models—in mitigating moral residue and fostering long‐term professional resilience. Ultimately, the goal of future inquiry must be to bridge the gap between acknowledging moral distress and engineering an ethically sustainable work environment.

## Author Contributions

F.S.N.R., N.R., and M.N. contributed to data collection, initial coding, and ongoing analysis. H.A. led the final manuscript preparation and the structured presentation of the findings. All authors have read and approved the final manuscript.

## Funding

The authors have nothing to report.

## Conflicts of Interest

The authors declare no conflicts of interest.

## Supporting information


**Data S1:** COREQ (COnsolidated criteria for REporting Qualitative research) checklist.

## Data Availability

The data that support the findings of this study are available from the corresponding author upon reasonable request.
